# Sanitary Crisis, Civilizational Crisis

**DOI:** 10.1177/1206331220938617

**Published:** 2020-08

**Authors:** Michel Maffesoli

**Affiliations:** 1 Institut Universitaire de France, Paris, Île-de-France, France; 2 La Sorbonne, Paris, France

**Keywords:** civilizational crisis, post-modernity, pandemic, solidarity, tragic

## Abstract

Sanitary Crisis, Civilizational Crisis is the translation of Michel Maffesoli’s *Crise sanitaire, crise civilisationnelle*. This paper can be taken as his pronouncement on the civilizational crisis that the COVID-19 pandemic acutely reveals. Maffesoli’s text urges one to see beyond secondary causes or dramatic representations of the pandemic as a sanitary crisis, and to consider the primary, and tragic, causes of this event, understood as a crisis that marks the exhaustion of the logic of modernity. Following from a longstanding critique of the decadence of modernity and, by extension, of an “official society” ordered and controlled by an out-of-touch and morbid elite, Maffesoli makes unequivocally clear that this global pandemic is a direct consequence of a globalized progressivist, economicist, and utilitarian civilizational paradigm. The paper takes up the task of reflecting on how relationality, being-together, and being-with, can be thought in our current moment of civilizational crisis.

Beyond our moods, worries, convictions, reactions, consent, and everything belonging to the realm of opinion, one mustn’t lose sight of the essential. That is to say, beyond appearances, and what the poet beautifully calls “the sloshing noise of secondary causes,” a return to the being of things. Short of “meditations,” of these evidences poured out *ad nauseam* by the intelligentsia, a return to what is immediately evident. It’s what folk wisdom cuttingly expresses: everything passes, everything breaks, everything tires!

In terms of the end of a modernity reaching the end of the race; saturation of a set of values that are more and more obsolete.

Let us remember, here, one of the etymologies of the term crisis: “*krisis*,” as in the judgement pronounced by what is being borne on what is dying. This, one too often forgets, reduces the crisis to its economic aspect. The simple dysfunction of what my late friend, Jean Baudrillard, named “the consumer society,” to which a few adjustments of the political order would not fail to serve as a corrective for the greater good of all.

Such is the way that one may understand the “sanitary crisis” as the modality of an ongoing social crisis, of a much more profound change of paradigm.

In other terms, sanitary crisis as visible expression of an invisible degeneracy. Degeneracy of an expired civilization. A civilization whose paradigm is no longer recognized. The matrix of the being-together has become barren (see Maffesoli, 2020).

Short-sighted rationalism may concede that this is a somewhat mysterious allegory, nay mystical. Yet history is not short of examples. In fact, they abound. I will content myself to recall of the grand correlative plague at the end of the Roman Empire. The famous “Antonine” plague of ce 190, while causing millions of deaths, also marked the beginning of the Roman decadence.

And what to say of the “Black Plague,” also known as the “Black Death,” that in the fourteenth century was the corollary of the end of the Middle Ages? The Renaissance was to succeed it. What the historians called the *Black Death* well expresses the mourning for a set of values that were no longer compatible with the new spirit of the time in gestation.

Let’s leave metaphor behind. Yet it has been a long-time since, along with a few others, suffering the thunderbolts of a frightened intelligentsia, I pointed to, underscored, and analyzed the *decadence of modernity*. The end of a world is no longer defended except by castes proud of their illusory superiority, crooning their fallacious rantings. This “official society” is more and more disconnected from real life, and, as such, is incapable of seeing the intellectual and political decay whose symptoms are more and more evident.

What decay, if not that of the myth of progress? I had shown since 1979 that correlated with the ideology of public service, this progressivism employed itself to justify the domination of nature, to the neglect of its primordial laws and in the construction of a world solely according to the principles of a rationalism whose morbid aspect appears more and more evident. A *Totalitarian Violence* (Maffesoli, 2008) of a progressivism as daft as it is destructive.

Once again, one must be bound to the *essential*. The nodal point of the progressive ideology is the ambition, even pretension, to solve everything, to improve everything in order to arrive at a perfect society, and at a potentially immortal human.

Whether we know it or not, the dialectic, thesis, antithesis, synthesis, is the dominant intellectual mechanism. The Hegelian concept of “sublation” (*Aufhebung*), is the master word of the progressive mythology. It is *stricto sensu*, a conception of a “dramatic” world, that is to say it rests on the capacity to find a solution, a resolution to that which can be a barrier to the perfection to come.

There is a formula proper to the nineteenth century that summarizes such a mythology well: each society only poses to itself the problems that it can resolve. Ambition is the pretension to master everything. It is the economy of salvation or the history of the Judaeo-Christian salvation by obedience that, in the big socializing systems of the nineteenth century, becomes “profane” [secular] and will inspire all of the political programs, both left and right.

Indeed, it is this *dramati* **c**, and therefore optimistic conception that is coming to an end. And, in the inexorable swing of human histories, it is “the tragic sentiment of life” (Miguel de Unanumo) that, once again, tends to prevail. The dramatic, I have claimed, is resolutely optimistic. The *tragic* is *aporic*, that is to say without solution. Life is what it is.

Rather than wanting to dominate nature, one attunes oneself to her following the popular adage, “Nature, to be commanded, must be obeyed.” Death, then, is no longer what one could overcome, but that to which one must be attuned.

This is what is restated, in a major key, by the “sanitary crisis.” The pandemic death is a symbol of the end of the optimism proper to modern progressivism. One can consider it as an expression of the mystical presentiment that the death of a civilization may be a deliverance, and, in its strong sense, the indication of a renaissance. It is an “index” that points to the continuity of an essential vitalism!

Possible death, a threat that is lived daily, a reality that cannot be denied, that can no longer be denied—death that, inexorably, one is obligated to tally— omnipresent, reminds us in its concreteness that an it is an order of things that is coming to an end.

What is concrete is, I repeat, *cum crescere*, what “grows with,” with an irrefragable real. And this real, could it be? Really? The death of this “order of things” that constituted the modern world!

Death of the dominant economicism, of this prevalence of the economic infrastructure of the “bourgeoisism” of the nineteenth century, cause and effect of a short-sighted utilitarianism. Despite the “consumer society,” Jean Baudrillard has clearly shown how all of social life was but a *Mirror of Production* (1975). *Being-together* is reduced to abstract “being,” uniquely preoccupied by material that we no longer master. One no longer possesses objects, one is possessed by them!

Death of a purely individualist conception of existence. Admittedly, the out-of-touch elites continue to emit clichés considering “contemporary individualism” and other twaddle of the sort. But the anguish of finitude, the reality of which can no longer be hidden, incites us, on the contrary, to helping one another, to sharing, to exchanging, to volunteering, and other values of the same sort that consumerist ideology believed had been surpassed.

Even “confined” in their apartment, it is interesting to note that patriotic songs, or those of the popular repertoire, are taken up together. And this is in order to conjure, collectively, the anguish proper to the feeling of finitude, and, as such, to express a solidarity in the face of death.

Even more flagrant, the sanitary crisis signals the death of globalization, the dominant value of an elite that, all tendencies confounded, remains beclouded by a market without limit, without border where, there again, the object prevails over the subject, the material over the spiritual.

Let us remember the judicious expression of the philosopher Georg Simmel, reminding us that the positive equilibrium of all social life is the accord that must exist between the “bridge and the door.” The bridge is necessary to the relation, and the door relativizes this relation in order to reach a harmony beneficial to all.

Excessive globalization is, it is hard to recognize, the heritage of the *Universalism* proper to the philosophy of the Enlightenment of the eighteenth century. The saturation of such a state of affairs will valorize localism, what the School of Palo Alto, in California, correctly named, “proxemia.” That is to say, the existing interaction between the natural and social environments.

What I called “Ecosophy,” the wisdom of the common house, or in more familiar terms, recognition that “*le lieu fait lien* ***”*** [the place makes the relation]. All things remind us that in encountering the well-known saying: “The urban air makes you free,” the archetypal formula for uprootedness, the natal glebe finds a renewed force and vigor that are undeniable.

*Dynamic rootedness* reminds us that, as with any plant, the human plant needs roots to grow with strength, accuracy, and beauty! Thus, in the face of an ever-so-present death, the need for the solidarity proper to a “communitarian ideal” returns, despite its continued stigmatization by some who foolishly tax this ideal of communitarianism.

Some? Whom are they? Simply, those who, having the power to say and do, continue to defend with tooth and nail the economism, individualism, globalism, and utilitarianism discussed above.

The consanguinity of elites is a thing of evidence. Their endogamy is mortifying. This incestuousness could not be more evident in the moral clichés in which oligarchs’ delight—clichés badly hiding their atavistic cult of money, their economist orthodoxy, and their celebration of an outdated scale of values. All of this through incantations: democracy, republican values, secularity, progressivism, etc., expressed in convoluted formulas where sharp minds and popular common sense readily identify amphibology and vicious circles. Stereotyped formulas translating only the essence of their practices and the basis of their profound desire for “*over-administration*” ensuring them an unsurpassable power over a hopelessly weakened people.

These elites have forgotten that to command is to serve. This adage best expresses social cohesion: *regnare servire est*. In short, the equilibrium that must exist between the force, *puissance*, of the instituting and the power of the instituted, that is, of economic, political, and social institutions.

They do not grasp that quotidian death, recalling our good memory, ineluctably signals the death of modern materialist civilization, that there will be what the sociologist Vilfredo Pareto justly called the turnover or “circulation of elites” (1901/1968).

A turnover that, with the help of the Internet, means the death of the verticality of power to the benefit of the horizontality of societal *puissance*. I often argued that postmodernity is nothing but the *synergy of the archaic and of technological development*. Or in another manner of saying, the return to sharing, exchange, solidarity, and other primary and fundamental values that the paranoia of modern elites believed, dialectics helping, could be “surpassed.”

The death of the utilitarian civilization where the social tie is a dominant *mechanics*, unable to identify the re-emergence of an *organic* solidarity. Organicity that esoteric thought names “*synarchy*.” Georges Dumézil (2016) clearly analyzed this by reminding us of the interaction and equilibrium existing, occasionally, between the “three social functions.”

The spiritual function, founding the political, the military, the juridical and arriving at societal solidarity. Hence, beyond the over-administration disconnected from the Real, it is such a holism that is reappearing today.

But considering one such *organic synarchy* requires that one may know how to enunciate it with words most pertinent to the times. It is amusing, though it would be best to say distressing, to read under the pen of editorialists in good graces that the situation is *dramatic*, and a few lines further they speak of its *tragic* aspect.

Plato’s formula is still current: “the fraud of words” is the ineluctable sign of an accomplished degeneration. The “dramatic” conception pertains to an elite that believes that they can find an opportune solution to everything. The “tragic,” on the contrary, accepts death. It knows, from an incorporated knowledge, knowledge specific to popular wisdom, how to live everyday death.

In this way, the *sanitary crisis*, leading to individual death, is the sign of a civilizational crisis—that of the death of the expired progressive paradigm that has had its day. Perhaps it is this which makes the tragic ambient, lived in the everyday, far from being morose, conscious that it is a resurrection that is under way. One where in being-together, in the being-with and in the visible social, the invisible spiritual will occupy a place of honor.
